# A Rare Case of Penoscrotal Webbing and Extensive Hernias: An Anatomical Report With Genetic Insights

**DOI:** 10.7759/cureus.47375

**Published:** 2023-10-20

**Authors:** Carley M Olson, Andrey Frolov, Yun Tan, John R Martin, Meadow Campbell

**Affiliations:** 1 Department of Surgery - Center for Anatomical Science and Education, Saint Louis University School of Medicine, Saint Louis, USA

**Keywords:** wound healing, muscular pathologies, anatomical variation, genetic screen, hernias, penoscrotal webbing

## Abstract

During a routine anatomical dissection of an 81-year-old male cadaver received through the Gift Body Program of Saint Louis University School of Medicine (SLU SOM), a massive bulging in the abdominal area was observed that was consistent with numerous hernia repairs noted in the donor’s self-reported medical history. Gross anatomical dissection of the cadaveric body revealed extensive herniation of portions of the small intestine and peritoneal sac along the costal margin and extending to the left aspect of the abdomen. Additionally, an uncircumcised phallus was buried within the suprapubic fat pad and demonstrated simple, grade III penoscrotal webbing (PSW), creating an impression of micropenis presence. To gain additional insights into the current case, analysis of the coding regions (exomes) of DNA procured from the body for putative genetic variants was performed using next-generation sequencing (NGS) technology. This analysis revealed 110 rare (minor allele frequency (MAF) ≤ 0.01), pathologic/deleterious genetic mutations. The most relevant variants to this case were the ones associated with male sexual development, *BMP1* and *BMP4*; connective tissue development, *COL3A1* and *COL5A3*; cilia morphogenesis and function, *DNAH5* and *MAPK15*; as well as hormonal homeostasis, *ESR1*. Direct involvement of *BMP1* both in male sexual development and hernia genesis makes it a strong candidate for linking the two pathologies, PSW and multiple hernias, observed in the present case. Yet the presence of a group of mutated genes linked to myopathies (*ITGA7*, *NRAP*, *POLM*, *SCN5A*, *XIRP2*) and muscular dystrophy (*ITGA7*) raises a question about the involvement of these muscular pathologies in hernia genesis and unsuccessful hernia repairs associated with the current case.

## Introduction

Male sexual development occurs under the intricate control of many genes. Mutations involving these specific genes that play a role in male sexual development can result in genitalia malformations. The condition of penoscrotal webbing (PSW) has been discussed under a variety of names, including webbed penis, penoscrotal fusion, penoscrotal pterygium, and penis palmatus [1]. PSW is a condition in which a fold of skin connecting the penile shaft to the scrotum obscures the penoscrotal angle making an otherwise normal-sized penile shaft appear smaller than it actually is [1]. The prevalence of PSW is unknown; however, one study of 5,881 newborns found that about 4% had the condition [1]. Congenital PSW frequently coexists with other congenital anomalies such as hypospadias, chordee, buried penis, or micropenis [2]. Due to the physical and psychological impact that this condition can have on males, many parents elect for surgical correction in infancy.

The anatomical organization of male external genitalia emerges during embryonic and fetal development, and understanding the developmental biology of external genitalia is critical for appreciating pathologies that lead to malformations of the penis and surgical repair of such malformations [3]. The exact etiology of PSW is still unknown, but there are two hypotheses that suggest potential causes. A delay or disturbance in the development of the preputial skin could result in a deficiency of the ventral penile skin or abnormal dartos band attachment that could explain the condition [4]. Another hypothesis includes an abnormal attachment of the skin due to an embryonic remnant of a cloacal membrane that may contribute to PSW [5].

A classification system of PSW has been proposed by El-Koutby et al. and is the most used method in describing this condition and for planning surgical correction. This classification system first divides the condition into the primary and secondary webbed penis [1]. The primary webbed penis is congenital and can be further broken down into simple or compound cases, while the secondary webbed penis is acquired post-circumcision due to an overly aggressive resection of the prepuce. Primary simple webbed penis is further subdivided into grades I-III based on where scrotal skin attaches along the penile shaft [1]. Grade I PSW extends to the proximal one-third of the penile shaft, grade II extends to the middle one-third of the penile shaft, and the most extreme grade III PSW extends to the distal one-third of the penile shaft [1].

A thorough review of the literature regarding PSW, hernias, and genetic variants linked to such pathologies revealed no previous reports of the co-occurrence of PSW and extensive hernias. Though the normal penoscrotal relationship is suggested to have a genetic basis, the literature lacks reports that support this claim [5]. Given the scarcity of information that could shed light on the etiology of PSW, we decided to address this shortcoming by performing a genetic screen to probe for single nucleotide variants in the coding regions of DNA extracted from this individual.

This article was previously presented as a meeting abstract at the 2023 Anatomy Connect AAA Annual Scientific Meeting on March 25, 2023.

## Case presentation

Anatomical characterization

**Figure 1:**
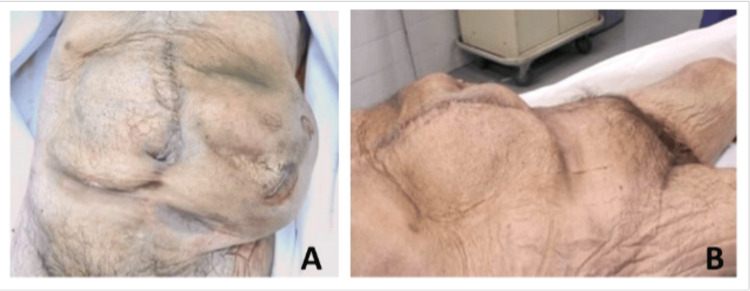
The abdominal appearance. A: An anterior view of the abdomen demonstrating a large midline scar and a large bulge off to the left aspect of the abdomen. B: A lateral view of the abdomen demonstrating a bulge above the pubic region.

The body of an 81-year-old male was received through the Gift Body Program of Saint Louis University School of Medicine (SLU SOM) with a signed informed consent of the donor. The self-reported medical history indicated at least seven hernia repairs, quadruple bypass heart surgery, irritable bowel syndrome, diverticulitis, and hypertension. This individual did have children, but it is not known if these children were biological. An external examination of the cadaver from the sternal angle to the feet was conducted. The upper limbs, neck, and head of this cadaver were absent as they were utilized elsewhere for surgical training purposes. Numerous scars were observed on the abdomen including a large midline scar in addition to a large bulge to the left aspect of the abdomen (Figure 1A). Another large bulge was present above the pubic region (Figure 1B). This appearance was later revealed to be a result of extensive hernias.

**Figure 2:**
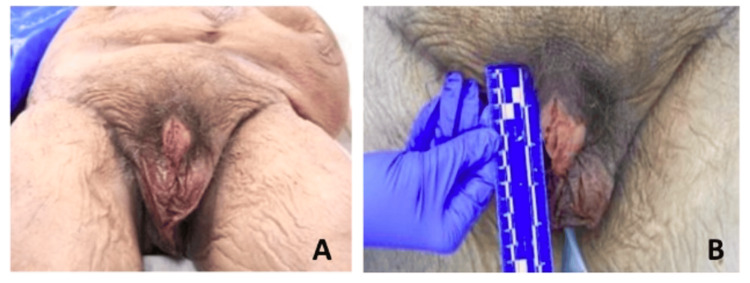
The external genitalia appearance. A: An anterior view of the pelvic region demonstrating an uncircumcised and diminished phallus. B: The phallus was measured at 5.33 cm (2.10 inches).

The phallus was uncircumcised (Figure 2A) with a penile length measured at 5.33 cm (2.10 inches) (Figure 2B). Due to the large hernia and fat deposits above the pubic symphysis, it was not possible to measure beginning at the pubic symphysis. Rather, the measurement was obtained by placing a ruler lateral to the penis to determine the length of the penile shaft that was exposed. The proximal portion of the penile shaft was palpated through the large bulge, but the entirety of the penile shaft was not visible or measured until after dissection.

**Figure 3:**
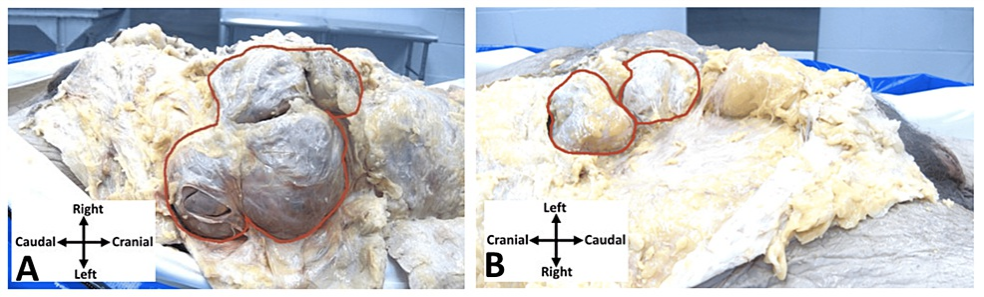
Abdominal hernias. A: Left lateral aspect of the abdomen demonstrating extensive hernias outlined in red. B: Right lateral aspect of the abdomen demonstrating extensive hernias outlined in red.

Anatomical dissection of the abdomen revealed extensive herniation of the small intestine within the peritoneal sac (Figure 3). These hernias traced the costal margin and extended to the left aspect of the abdomen. Due to the extent of the herniated abdominal contents and prior surgeries, it was difficult to determine the specific types of hernias present. Consultation with a general surgeon confirmed the presence of numerous incisional hernias along a large midline scar. Other hernias such as epigastric hernia and left primary Spigelian hernia could not be ruled out. Evidence of a previous umbilical hernia repair and left femoral hernia repair were observed.

**Figure 4:**
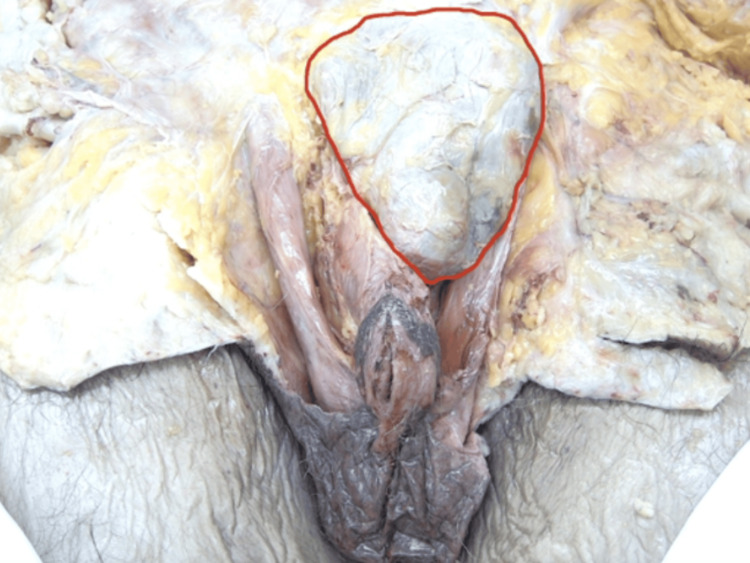
Suprapubic region. Herniation of a loop of small intestines through the hypogastric region of the anterior body wall outlined in red. The herniation was located superomedial to the inguinal ligament.

As removal of the skin continued inferiorly towards the region of the pubic symphysis, a large loop of small intestine was located within the suprapubic fat pad (Figure 4).

We believe that the presence of this hernia in addition to a large amount of suprapubic fat obscured the length of the shaft of the penis giving it a much smaller appearance than was present.

The anterior abdominal wall was removed and hernias that were incarcerated in the anterior abdominal wall were carefully freed. The entirety of the bowel was examined and revealed multiple stitches on the small intestine suggesting previous surgical procedures. Evidence of a left femoral hernia repair, including sutures and mesh, were observed and appeared to compress the left spermatic cord. The spermatic cord was dissected to reveal a normal ductus deferens that was traced into the pelvic cavity. Once in the pelvic cavity, the following structures were identified: ampulla of ductus deferens, seminal vesicles, ureters, prostate, urethra, and the related vasculature. The prostate was also intact and was located inferior to the bladder. All internal genital organs appeared normal. The testes were palpated within the scrotum to ensure that two were present, descended into the scrotum, and were of normal size measuring to be approximately 15.3 cm^3^ compared to the mean volume of 12-30 cm^3^ in normal males [6,7].

**Figure 5:**
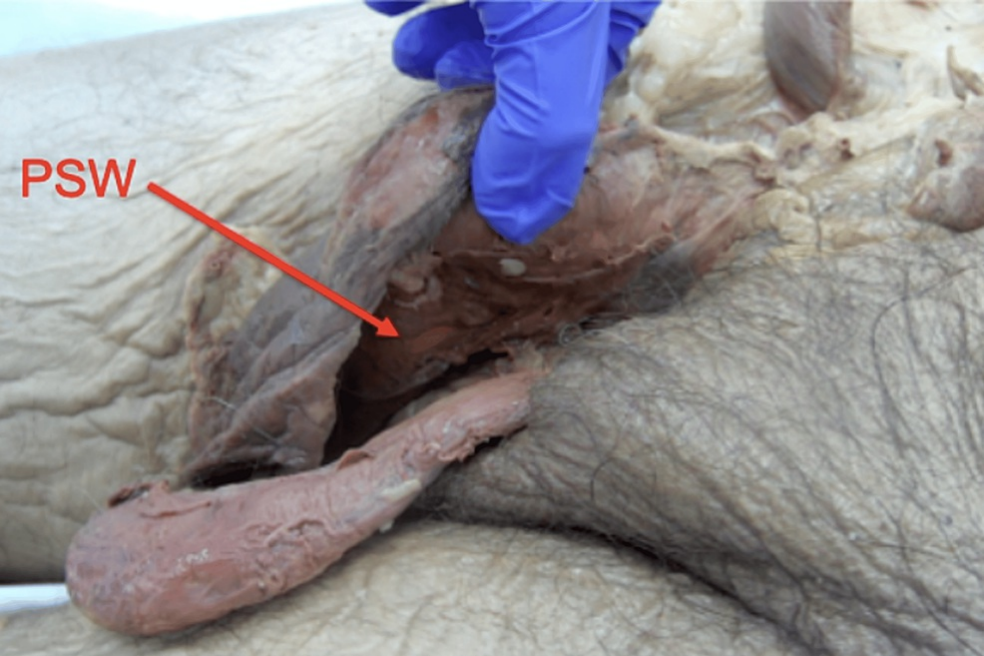
Dissection of penoscrotal webbing (PSW). Left lateral view of the PSW with the red arrow pointing to the webbing.

Lastly, a dissection of the perineum was conducted. A flap of tissue that appeared to be the scrotal septum was observed fusing the entire ventral length of the penile shaft to the scrotum (Figure 5).

Dissection of the scrotum revealed the uncircumcised phallus was buried within the suprapubic fat pad and accompanied by a simple, grade III PSW. All other male anatomical structures were observed to be normal.

Genetic screen

Given the uniqueness of this case of extensive abdominal hernias and penoscrotal webbing, a screen for the putative genetic variants was performed using next-generation sequencing (NGS) on the Illumina platform. The exome sequencing of DNA extracted from the embalmed cadaveric tissue revealed 110 rare (minor allele frequency (MAF) ≤ 0.01), pathologic/deleterious genetic variants (Table 1).

**Table 1:** Complete list of deleterious (pathologic) genetic variants associated with the current case.

Gene	Protein Function
ABCA10	ATP Binding Cassette Subfamily A Member 10. The membrane-associated protein encoded by this gene is a member of the superfamily of ATP-binding cassette (ABC) transporters.
ADAM18	ADAM Metallopeptidase Domain 18. This gene encodes a member of the ADAM family that has been implicated in a variety of biological processes involving cell-cell and cell-matrix interactions such as fertilization, muscle development, and neurogenesis.
AMACR	Alpha-Methylacyl-CoA Racemase.
ATP10B	ATPase Phospholipid Transporting 10B.
B3GNT4	UDP-GlcNAc:BetaGal Beta-1,3-N-Acetylglucosaminyltransferase 4.
BMP1	Bone Morphogenetic Protein 1. Participates in cartilage and bone formation, muscle growth, wound healing, and tissue repair.
BMP4	Bone Morphogenetic Protein 4. Provides a delicate balance between apoptosis and proliferation of the genital tubercle. Involved in cartilage and bone formation, prostate gland, ureteric bud morphogenesis.
BTN3A3	Butyrophilin Subfamily 3 Member A3.
CDHR4	Cadherin Related Family Member 4.
CELA3A	Chymotrypsin Like Elastase 3A.
CEP162	Centrosomal Protein 162. Involved in cilium assembly. Located in axonemal microtubule; centriole; and centrosome.
CEP41	Centrosomal Protein 41. This gene encodes a centrosomal and microtubule-binding protein which is predicted to have two coiled-coil domains and a rhodanese domain.
CLHC1	Clathrin Heavy Chain Linker Domain Containing 1.
CNTN5	Contactin 5.
COL3A1	Collagen Type III Alpha 1 Chain. Collagen type III occurs in connective tissues with type I collagen. Involved in fibrillogenesis and wound healing.
COL5A3	Collagen Type V Alpha 3 Chain. This gene encodes an alpha chain for one of the low abundance fibrillar collagens. Type V collagen is found in tissues containing type I collagen and appears to regulate the assembly of heterotypic fibers composed of both type I and type V collagen. Mutations in this gene are responsible for the symptoms of a subset of patients with Ehlers-Danlos syndrome type III.
CPA3	Carboxypeptidase A3.
CST4	Cystatin S.
CTNNBIP1	Catenin Beta Interacting Protein 1. The protein encoded by this gene is a negative regulator of the WNT signaling pathway.
CTNND2	Catenin Delta 2.
DDX28	DEAD-Box Helicase 28. DEAD box proteins, characterized by the conserved motif Asp-Glu-Ala-Asp, are putative RNA helicases. They are implicated in a number of cellular processes in addition to embryogenesis, spermatogenesis, cellular growth and division.
DGAT2	Diacylglycerol O-Acyltransferase 2.
DMRTA1	Doublesex- And Mab-3-Related Transcription Factor A1. Enables sequence-specific double-stranded DNA binding activity. Predicted to be involved in germ cell development, regulation of transcription by RNA polymerase II, and sex differentiation.
DNAH5	Dynein Axonemal Heavy Chain 5. Cilium assembly, cilium movement, determination of left/right asymmetry.
DNAH7	Dynein Axonemal Heavy Chain 7. component of the inner dynein arm of ciliary axonemes.
DNM3	Dynamin 3.
DPH1	Diphthamide Biosynthesis 1.
EIF3L	Eukaryotic Translation Initiation Factor 3 Subunit L.
ELF5	E74 Like ETS Transcription Factor 5.
ESR1	Estrogen Receptor 1. Plays a role in growth, metabolism, sexual development, gestation, and other reproductive functions. Estrogen controls many cellular processes including growth, differentiation and function of the reproductive system.
FANCI	Fanconi Anemia Complementation Group I.
FAT4	FAT Atypical Cadherin 4.
FN1	Fibronectin 1. This gene encodes fibronectin, a glycoprotein presents in a soluble dimeric form in plasma, and in a dimeric or multimeric form at the cell surface and extracellular matrix. Involved in cell adhesion and migration processes including embryogenesis, wound healing, blood coagulation, host defense, and metastasis. Participates in the regulation of type I collagen deposition by osteoblasts.
GABRD	Gamma-Aminobutyric Acid Type A Receptor Subunit Delta.
HPR	Haptoglobin-Related Protein.
HPS3	HPS3 Biogenesis of Lysosomal Organelles Complex 2 Subunit 1.
HS6ST1	Heparan Sulfate 6-O-Sulfotransferase 1.
HSF4	Heat Shock Transcription Factor 4.
INA	Internexin Neuronal Intermediate Filament Protein Alpha.
IREB2	Iron Responsive Element Binding Protein 2.
ITGA7	Integrin Subunit Alpha 7. The protein encoded by this gene is expressed in skeletal and cardiac muscles and may be involved in differentiation and migration processes during myogenesis. Defects in this gene are associated with congenital myopathy.
KIF25	Kinesin Family Member 25.
KRT40	Keratin 40.
KRTAP26-1	Keratin Associated Protein 26-1.
LRIG2	Leucine Rich Repeats And Immunoglobulin Like Domains 2.
LRP1B	LDL Receptor Related Protein 1B.
LRRC14B	Leucine Rich Repeat Containing 14B.
LRRC71	Leucine Rich Repeat Containing 71.
LZTS2	Leucine Zipper Tumor Suppressor 2. function in transcription regulation and cell cycle control. This family member can repress beta-catenin-mediated transcriptional activation and is a negative regulator of the WNT signaling pathway.
MAPK15	Mitogen-Activated Protein Kinase 15. Regulates primary cilium formation and the localization of ciliary proteins involved in cilium structure, transport, and signaling. Expression is highest in the gonads.
MBL2	Mannose Binding Lectin 2.
MCM4	Minichromosome Maintenance Complex Component 4.
MDN1	Midasin AAA ATPase 1. Predicted to enable ATP binding activity.
NEDD8-MDP1	NEDD8-MDP1 Readthrough.
MICALL2	Molecule Interacting With CasL-Like 2.
MUC20	Mucin 20, Cell Surface Associated.
NAV1	Neuron Navigator 1.
NCOR2	Nuclear Receptor Corepressor 2.
NDUFV1	NADH: Ubiquinone Oxidoreductase Core Subunit V1.
NRAP	Nebulin Related Anchoring Protein. Located in fascia adherens, muscle tendon junction, and myofibril. Predicted to enable actin filament binding activity and muscle alpha-actinin binding activity.
OR1E2	Olfactory Receptor Family 1 Subfamily E Member 2.
OR2C1	Olfactory Receptor Family 2 Subfamily C Member 1.
OR51G2	Olfactory Receptor Family 51 Subfamily G Member 2.
OR5F1	Olfactory Receptor Family 5 Subfamily F Member 1.
OTOP1	Otopetrin 1.
P2RX5	Purinergic Receptor P2X 5.
PCDHGA1	Protocadherin Gamma Subfamily A, 1.
PCDHGA11	Protocadherin Gamma Subfamily A, 11.
PCDHGA7	Protocadherin Gamma Subfamily A, 7.
PHYHD1	Phytanoyl-CoA Dioxygenase Domain Containing 1.
PLAU	Plasminogen Activator, Urokinase.
PLEKHG1	Pleckstrin Homology and RhoGEF Domain Containing G1.
PLEKHG4	Pleckstrin Homology and RhoGEF Domain Containing G4.
PLK4	Polo Like Kinase 4.
PMP22	Peripheral Myelin Protein 22.
POLM	DNA Polymerase Mu. The associated protein is involved in DNA polymerase activity and is linked to congenital myopathy 2A.
PRKN	Parkin RBR E3 Ubiquitin Protein Ligase.
RDX	Radixin.
RHPN2	Rhophilin Rho GTPase Binding Protein 2.
SCN5A	Sodium Voltage-Gated Channel Alpha Subunit 5. The protein encoded by this gene is an integral membrane protein and is responsible for the initial upstroke of the action potential.
SEPTIN10	Septin-10.
SIGLEC6	Sialic Acid Binding Ig Like Lectin 6.
SLC10A2	Solute Carrier Family 10 Member 2.
SLC30A5	Solute Carrier Family 30 Member 5.
SRA1	Steroid Receptor RNA Activator 1.
ST8SIA6	ST8 Alpha-N-Acetyl-Neuraminide Alpha-2,8-Sialyltransferase 6.
STAB2	Stabilin 2.
STXBP4	Syntaxin Binding Protein 4. Enables syntaxin binding activity.
TCOF1	Treacle Ribosome Biogenesis Factor 1.
TECPR2	Tectonin Beta-Propeller Repeat Containing 2.
TMPRSS4	Transmembrane Serine Protease 4.
TMPRSS6	Transmembrane Serine Protease 6.
TNFRSF8	TNF Receptor Superfamily Member 8.
TRIM11	Tripartite Motif Containing 11.
TRIM16	Tripartite Motif Containing 16.
TRIM65	Tripartite Motif Containing 65.
URB1	URB1 Ribosome Biogenesis Homolog.
WRNIP1	WRN Helicase Interacting Protein 1.
XIRP2	Xin Actin Binding Repeat Containing 2. Enables actin filament binding activity. Predicted to be involved in actin cytoskeleton organization and predicted to act upstream of or within muscle tissue morphogenesis.
YIF1A	Yip1 Interacting Factor Homolog A, Membrane Trafficking Protein.
ZDBF2	Zinc Finger DBF-Type Containing 2.
ZNF559	Zinc Finger Protein 559.
ZNF611	Zinc Finger Protein 611.
ZNF695	Zinc Finger Protein 695.
ZNF732	Zinc Finger Protein 732.

Seven genetic variants, *BMP1*, *BMP4*, *COL3A1*, *COL5A3*, *MAPK15*, *DNAH5*, and *ESR1*, could be linked directly to this case (Table 2) due to their involvement in wound healing and male sex organ development. 

**Table 2:** Selected rare deleterious/pathological variants associated with the current case.

Gene	Protein Function	Variant
BMP1	Bone morphogenetic protein 1. Participates in cartilage and bone formation, muscle growth, wound healing and tissue repair.	NM_001199:exon9:c.G1112A:p.R371H NM_006129:exon9:c.G1112A:p.R371H
BMP4	Bone morphogenetic protein 4. Provides a delicate balance between apoptosis and proliferation of the genital tubercle. Involved in cartilage and bone formation. Prostate gland, ureteric bud morphogenesis.	NM_001347914:exon2:c.C205T:p.R69C NM_001347915:exon2:c.C16T:p.R6C NM_001347912:exon3:c.C346T:p.R116C
COL3A1	Collagen Alpha-1(III) Chain. Collagen type III occurs in connective tissues with type I collagen. Involved in fibrillogenesis and wound healing.	NM_000090:exon29:c.C2002A:p.P668T
COL5A3	Collagen Type V Alpha 3 Chain. This gene encodes an alpha chain for one of the low abundance fibrillar collagens. Type V collagen is found in tissues containing type I collagen and appears to regulate the assembly of heterotypic fibers composed of both type I and type V collagen. Mutations in this gene are responsible for the symptoms of a subset of patients with Ehlers-Danlos syndrome type III.	NM_015719:exon43:c.C3148T:p.P1050S
MAPK15	Mitogen-Activated Protein Kinase 15. Regulates primary cilium formation and the localization of ciliary proteins involved in cilium structure, transport, and signaling. Expression is highest in the gonads.	NM_139021:exon5:c.G407A:p.R136Q
DNAH5	Dynein axonemal heavy chain 5. Cilium assembly, cilium movement, determination of left/right asymmetry.	NM_001369:exon35:c.A5848G:p.T1950A NM_001369:exon10:c.T1206A:p.N402K
ESR1	Estrogen Receptor 1. Plays a role in growth, metabolism, sexual development, gestation, and other reproductive functions. Estrogen controls many cellular processes including growth, differentiation and function of the reproductive system.	NM_000125:exon4:c.C805T:p.R269C NM_001328100:exon4:c.C286T:p.R96C NM_001291230:exon5:c.C811T:p.R271C NM_001291241:exon5:c.C802T:p.R268C
ITGA7	Integrin Subunit Alpha 7. The protein encoded by this gene is expressed in skeletal and cardiac muscles and may be involved in differentiation and migration processes during myogenesis. Defects in this gene are associated with congenital myopathy.	NM_001144996:exon8:c.C1211T:p.A404V NM_001144997:exon8:c.C920T:p.A307V NM_002206:exon8:c.C1199T:p.A400V NM_001367993:exon9:c.C872T:p.A291V
NRAP	Nebulin Related Anchoring Protein. Located in fascia adherens, muscle tendon junction, and myofibril. Predicted to enable actin filament binding activity and muscle alpha-actinin binding activity.	NM_001322945:exon34:c.G3976A:p.D1326N NM_006175:exon34:c.G3979A:p.D1327N NM_001261463:exon35:c.G4084A:p.D1362N
POLM	DNA Polymerase Mu. The associated protein is involved in DNA polymerase activity and is linked to congenital myopathy 2A.	NM_001284330:exon5:c.G659C:p.G220A
SCN5A	Sodium Voltage-Gated Channel Alpha Subunit 5. The protein encoded by this gene is an integral membrane protein and is responsible for the initial upstroke of the action potential.	NM_000335:exon16:c.A2450G:p.K817R
XIRP2	Xin Actin Binding Repeat Containing 2. Enables actin filament binding activity. Predicted to be involved in actin cytoskeleton organization and predicted to act upstream of or within muscle tissue morphogenesis.	NM_001199144:exon7:c.T9784C:p.W3262R NM_152381:exon9:c.T10450C:p.W3484R

## Discussion

The uniqueness and significance of the present case is severalfold. First, the abnormality of the external genitalia in the current case can be categorized as primary simple, grade III PSW according to the classification system proposed in El-Koutby et al. [1]. This condition is rare and surgical correction is typically performed in infancy [1]. Given the age of the individual's body examined in this report (81 years) and the observation that no other major genitalia abnormalities developed, this could define the current case as isolated PSW thereby providing a unique opportunity to gain insights into the respective molecular mechanisms.

The results presented in this case support clinical observations pointing toward PSW being polygenic in nature and provide important insights into its etiology. Indeed, the respective genetic screen identified mutations in four genes that could be closely linked to the development of human external genitalia: *BMP4*, *DNAH5*, *MAPK15*, and *ESR1* (Table 2). There are numerous molecular mechanisms that govern the development of the external male genitalia including the penis and scrotum. In mice, the Sonic hedgehog (*SHH*) signaling pathway has been identified as a key regulator during initiation of morphologic differentiation of the genital tubercle and its outgrowth [8]. The differentiation of the genital tubercle after the initiation of *SHH* signaling leads to the upregulation of *BMP4*, *HOXA13*, and *HOXD13*, which would work to provide a delicate balance between apoptosis and proliferation. This balance is necessary for the proper differentiation of the genital tubercle and its dysregulation can result in abnormalities involving genital tubercle elongation and the spatiotemporal balance between apoptosis and proliferation [9].

*DNAH5* and *MAPK15* are involved in the formation and maintenance of cilia, including the primary cilia [10,11], which serves as a platform shared by *SHH* [12], *FGF* receptor [13], and *WNT* signaling pathways [14]. Therefore, in this case, the spatiotemporal dysregulation of the primary cilia formation and function may result in the aberrant *SHH*, *FGF*, and *WNT* signaling. This, along with the mutation in *BMP4*, which might negatively affect its proapoptotic function, could disrupt a dynamic equilibrium between cell proliferation and apoptosis [9], thereby skewing the developmental process of human male external genitalia resulting in PSW. There is no evidence to suggest that PSW affects a male’s fertility, though PSW can make penetrative intercourse more difficult or painful. Also, during prenatal and postnatal periods, the development of external genitalia is influenced by steroid hormones [15]. Specifically relevant to the current case, the expression of *ESR1* at the penoscrotal junction is suggested to play a role in the etiology of penile webbing [16]. The detected mutation in *ESR1* in this individual supports this notion.

Second, the present case is unique because there have been no previous reports of an individual with PSW also displaying such extensive abdominal hernias. PSW is considered to fit within the spectrum of penoscrotal transposition (PST) [17]. Depending on the severity of the PST, associated anomalies can be expected, especially of the gastrointestinal tract [18]. Though previously reported associated anomalies involving the gastrointestinal tract include omphalocele and gastroschisis, the extensive hernias seen in the current case cannot be overlooked. Though the etiology of hernias is multi-factorial, it is prevalent that alterations in the ratio of collagen type I to III can impact the likelihood of an individual developing various types of hernias including direct inguinal, recurrent inguinal, incisional, and recurrent incisional hernias [19-21]. Mutations specifically in *COL3A1* have also been linked to both hiatal hernias and bilateral inguinal hernias [22,23]. Patients with an inguinal hernia demonstrated a systemic and persistent type V collagen turnover alteration. This imbalance of collagen metabolism may be involved in the development of inguinal hernias. Unfortunately, the quality of the cadaveric tissue in the present case was incompatible with immunohistochemical staining. However, the study subject had a mutation in *COL5A3*, a gene coding for type V collagen in addition to bilateral inguinal hernia repair [24], which would be consistent with the impairment of type V collagen function and turnover. Individuals with mutations in *BMP1* have also been found to have large umbilical hernias [25]. With PSW typically corrected surgically in childhood, it is possible that these two anomalies, PSW and extensive abdominal hernias, are linked but have not been previously reported.

Third, the current case is unique because of the presence of numerous hernia repairs that resulted in extensive incisional hernias, primarily due to an aberrant wound-healing process. The latter is supported by the detected mutations in three genes: *COL3A1*, *COL5A3*, and *BMP1*. *COL3A1* encodes collagen type III alpha 1 chain while *COL5A3* encodes type V collagen, which is found in tissues containing type I collagen and regulates the assembly of heterotypic fibers composed of both type I and type V collagen. Because an aberrant collagen I to collagen III ratio can compromise skin repair and make it more vulnerable to wound recurrence, it is possible that these *COL3A1* and *COL5A3* mutations are responsible for the extensiveness of the incisional hernias in this individual. Also, given the importance of the extracellular matrix (ECM) in wound repair, the reported mutation in *BMP1* is also of significant interest. Indeed, mutations in this gene have been reported to result in impaired production of type I collagen, which in this case would also compromise the integrity of the ECM [25].

Fourth, the presence of a group of mutated genes linked to myopathies (*ITGA7*, *NRAP*, *POLM*, *SCN5A*, *XIRP2*) and muscular dystrophy (*ITGA7*) (Table 2) raises an important question regarding their involvement in the hernia development and the incomplete hernia repair in the present case. Indeed, a close association between myopathy and hernias including inguinal [26,27], hiatal [28], diaphragmatic [29], and experimental incisional [30] types have been previously reported. Additionally, either pre-existing [31] or developed local muscular dystrophy in patients with hernias [32,33] could complicate surgical procedures for hernia repair. More importantly, the pre-existing muscular dystrophy could contribute to hernia genesis as it has been previously shown for double ipsilateral inguinal hernia [34]. Therefore, the genetic and physiological testing of patients for pre-existing myopathies and muscular dystrophy should be considered prior to surgical hernia repairs, particularly for individuals with multiple hernias and/or a history of previous unsuccessful repair attempts.

## Conclusions

The increasing trend in the incidence of genital malformations and the decline in human male reproductive health have attracted much concern in recent years. Though the knowledge of the impact that genetics has on the development of external and internal genitalia has improved in recent decades, the exact genetic underpinning is yet to be identified. However, direct involvement of *BMP1* both in male sexual development and hernia genesis makes it a strong candidate for linking the two pathologies together, PSW and multiple hernias, observed in the present case.

In summary, the current case has a high clinical and educational value because it not only describes the co-existence of two unique anatomical abnormalities, PSW and multiple hernias, but also provides important insights into their potential common genetic underpinnings.
